# Glucocorticoids decreased Cx43 expression in osteonecrosis of femoral head: The effect on proliferation and osteogenic differentiation of rat BMSCs

**DOI:** 10.1111/jcmm.16103

**Published:** 2020-11-17

**Authors:** Xin Zhao, Mohammed Alqwbani, Yue Luo, Changjun Chen, Ge A, Yang Wei, Donghai Li, Qiuru Wang, Meng Tian, Pengde Kang

**Affiliations:** ^1^ Department of Orthopaedics Surgery West China Hospital Sichuan University Chengdu China; ^2^ Neurosurgery Research Laboratory West China Hospital Sichuan University Chengdu China

**Keywords:** bone marrow mesenchymal stem cells, connexin43, dexamethasone, ERK1/2 signalling pathway, osteonecrosis of femoral head

## Abstract

Glucocorticoid (GC)‐induced osteonecrosis of the femoral head (GC‐ONFH) is considered as one of the most serious side effects of long‐term or over‐dose steroid therapy. However, the underlying cause mechanisms are still not fully investigated. We firstly established a rat model of GC‐ONFH and injected lipopolysaccharide (LPS) and methylprednisolone (MPS). We found that the expressions of Cx43, Runx2, ALP and COLⅠ were more decreased than the normal group. Secondly, the isolated rat bone marrow stem cells (BMSCs) were treated with dexamethasone (Dex) in vitro, and the expressions of Cx43, Runx2, ALP and COLⅠ were decreased significantly. Moreover, the results of immunofluorescence staining, alizarin red staining, EdU assay and CCK8 showed that the osteogenic differentiation and the proliferation capacity of BMSCs were decreased after induced by Dex. A plasmid of lentivirus‐mediated Cx43 (Lv‐Cx43) gene overexpression was established to investigate the function of Cx43 in BMSCs under the Dex treatment. Findings demonstrated that the proliferation and osteogenic differentiation abilities were enhanced after Lv‐Cx43 transfected to BMSCs, and these beneficial effects of Lv‐Cx43 were significantly blocked when PD988059 (an inhibitor of ERK1/2) was used. In conclusion, the overexpression of Cx43 could promote the proliferation and osteogenic differentiation of BMSCs via activating the ERK1/2 signalling pathway, which provide a basic evidence for further study on the detailed function of Cx43 in GC‐ONFH.

## 1 INTRODUCTION

Osteonecrosis of the femoral head (ONFH) is a common disease in orthopaedics, and its debilitating effects could eventually lead to articular cartilage collapse and subsequent osteoarthritis.^1^, ^2^ The excessive use of glucocorticoid (GC) was regarded as one of the most common pathogenic factor of ONFH,^3^, ^4^ and the inhibition of the osteogenic differentiation of bone marrow stem cells (BMSCs) was considered to be an underlying cause for ONFH, which could inhibit the formation of bone tissues.^5^ Therefore, strategies that could promote BMSCs' proliferation and osteogenesis process might trigger the repairing process and decrease the progression of GC‐induced ONFH (GC‐ONFH).

Bone marrow stem cells are very important cells in the pathophysiology process of ONFH that are easily acquired and obtained due to limited ethical restrictions. They also have the capacity of self‐renewal and multilineage differentiation.^6^, ^7^, ^8^ Therefore, they are widely recommended to be used in cell therapy and tissue regeneration medicine. It is reported that GC overdose could decrease the osteogenic differentiation of BMSCs,^9^, ^10^ despite the pathophysiological mechanism of GC‐ONFH still unknown.

A gap junction channel consists of two hemichannels and each one formed by six connexins—transmembrane proteins.^11^, ^12^, ^13^ It was reported that connexins play a vital role in tissue homeostasis,^14^ in the regulation of cell proliferation and growth, and in cell differentiation and development.^15^ There are at least 21 kinds of connexins that existing in various tissues or cell specificity which and were verified by a spectrum of homologs.^16^, ^17^ Among these connexins, connexin43 (Cx43) is considered as a primary component of gap junctions in haematopoietic tissue.^17^ In osteoblasts and osteocytes, Cx43 plays an important role in the transmission of hormone mechanical load and the signalling induction by Prostaglandin E2 or growth factors.^18^, ^19^ In addition, several studies suggested that Cx43 was closely associated with the osteogenesis and osteoblast function.^20^, ^21^ Furthermore, studies showed that the correlative function of Cx43 lies in regulating ERK activity and consequently regulates Runx2, which is an essential transcription factor for osteoblast differentiation.^22^, ^23^ It is reported that high dose of dexamethasone (Dex) exposure could inhibit the osteogenesis of bone tissue and the expression of Cx43.^24^ Gang Liu and colleagues' findings indicated that Cx43 was involved in the pathogenesis of the early stages of GC‐ONFH,^24^ they found that Cx43 expression was significantly decreased in rabbit GC‐ONFH model compared with control group. However, the mechanism of how Cx43 regulates the differentiation of BMSCs is still not clear during GC‐ONFH development. Therefore, the objective of this study is to explore the function of Cx43 in osteogenic differentiation and proliferation of BMSCs under the disease of GC‐ONFH.

The present study investigated and analysed the relationship between the expression quantity of Cx43 and osteogenesis‐related proteins in vivo and in vitro. We found that Cx43 expression level decreased significantly in both the GC‐ONFH model and BMSCs after treated with high concentration of steroid, which were not different from the results of the expressions of the following osteogenesis‐related proteins: Runx2, alkaline phosphatase (ALP), and collagen type Ⅰ (COLⅠ). In addition, the expression of proliferation related proteins including PCNA and CDK4 were consistent with the expression of Cx43 in BMSCs that were treated with Dex. Moreover, over‐expression of Cx43 significantly increased osteogenic differentiation as well as the expression of Runx2, ALP, and COLⅠ. In the meanwhile, the proliferation ability was enhanced which verified by CCK8 and EdU staining, as well as the increased expression of PCNA and CDK4. These beneficial effects of Lv‐Cx43 were significantly blocked by PD98059 (an inhibitor of ERK1/2). Therefore, these results have demonstrated that Cx43 may play an important role in the pathophysiological process of BMSCs under the condition of GC‐ONFH, and over‐expression of Cx43 could effectively enhance the proliferation and osteogenic differentiation of BMSCs via up regulating the ERK1/2 signalling pathway. These findings concluded that Cx43 might become an important therapeutic target for GC‐ONFH and could be used for further studies.

## 2 MATERIALS AND METHODS

### 2.1 Animals

The guidelines of the National Institution of Health on the humane use and care of laboratory animals were applied on all the animal experiments in this study and the protocols were agreed upon by the Institutes Animal Care and Use Committee of West China Medical School of Sichuan University. A total of 24 adult male Sprague‐Dawley (SD) rats (450‐500 g) were housed for 6 weeks at Animal Center of Sichuan University and prepared to be used for animal experiments. Another Six of 2‐week‐old male SD rats were also prepared for acquiring and isolating BMSCs.

#### 2.1.1 Establishment of GC‐ONFH Model on rat

The 24 rats were randomly divided into two groups: ONFH model group (12 rats) and normal control group (12 rats). Each group was also randomly divided into two groups including 4 weeks (6 rats) and 6 weeks (6 rats) after the first MPS or saline injection. Firstly, the rats in the model group were injected with 10 μg/kg of lipopolysaccharide (LPS; Sigma) intravenously. After 24 hours, they were injected once a day intramuscularly with 40 mg/kg of methylprednisolone (MPS) for the following three days. The rats in the control group were also given the same volume of normal saline injections and kept under identical conditions.

#### 2.1.2 Samples collection

Based on results from our previous study,^25^ all the rats were killed at four or six weeks after the first MPS injection, and the bilateral femoral heads were dissected. All the collected samples were washed twice with ice phosphate buffer saline (PBS), then the left femoral heads were put in liquid nitrogen for further study by western blot method, and the right femoral heads were fixed with 10% formalin for further study, such as H&E staining.

#### 2.1.3 Haematoxylin & eosin staining

Haematoxylin & eosin (H&E) staining was performed to assess the histomorphological changes in the femoral heads and observe the rate of empty osteocytes lacunae and the destruction degree of bone trabecula, according to the method that was reported in our previous study.^26^ Firstly, the femoral heads that were fixed with 10% formalin for 24 hours, were decalcified in Ethylene diamine tetraacetic acid (EDTA, 10%) solution for four weeks, and then embedded in paraffin. Secondly, the Samples were cut into 3 μm thick sections and then were deparaffinized in xylene and dehydrated by ethyl alcohol. Finally, the samples were stained by haematoxylin and eosin stainings. Each slice was observed under the microscope and five random fields were chosen to calculate the average empty osteocytes lacunae. Then the final rate was calculated by Image‐Pro Plus 6.0 (Media Cybernetics, Baltimore, MD, USA).

#### 2.1.4 Western blot analysis

After mechanically grinding the tissues of the samples in mortar, RIPA lysis buffer (990 μL RIPA and 10 μL phenylmethylsulfonyl fluoride) was added to the tissues on ice. After 30 minutes, the supernatants were put into a new 1.5 mL centrifuge tube and centrifuged at 4°C for 15 minutes at 17900 *g*, and then the undissolved materials were abandoned. The protein concentrations of the supernatants were examined by BCA Protein Assay kit (Beyotime). 10% or 12% SDS‐PAGE method was used to separate and obtain the targeted proteins (20 μg), then we used a transfer unit (Bio‐Rad) to put the proteins onto PVDF membranes (0.45 μm; Millipore). the membranes were then kept in PBS solution containing 5% skim milk powder for two hours at 25°C. Then we added primary antibodies to these membranes for overnight at 4°C; primary antibodies included Cx43 (rabbit, 1:1000; abcam, ab11370), Runx2 (rabbit, 1:1000; Santa Cruz, sc‐390715), ALP (rabbit, 1:500; Santa Cruz, sc‐365765), COLⅠ (rabbit, 1:1000; Boster Bio, bs‐10423R), PCNA (rabbit, 1:1000; Proteintech, 10205‐2‐AP), CDK4 (mouse, 1:1000; Santa Cruz, sc‐23896), ERK1/2 (rabbit, 1;1000; abcam, ab17942), p‐ERK1/2 (rabbit, 1:1000; abcam, ab214362), β‐actin (mouse, 1:1000; ZSBIO, TA‐09) and GAPDH (mouse, 1:1000; Sigma‐Aldrich, SAB2108266). After washing the membranes with PBST, secondary antibodies (1:10 000; ZSBIO, ZDR‐5306/5307) were incubated for two hours and with membranes that were washed with PBST, then were being washed again with PBST three times. Finally, ECL method was used to examine the membranes and Quantity One software (Bio‐Rad) was used to analyse the results.

### 2.2 Experiments in vitro

#### 2.2.1 Cells isolation, culture and identification

A total of 1‐2 mL bone marrow samples were taken with a heparinized syringe from the lateral tibial tubercle of six newborn male SD rats (2‐weeks‐old). Low‐glucose DMEM (low‐DMEM) containing 10% foetal bovine serum (FBS) (Gibco) was used to wash the samples of bone marrow, we then centrifuged the samples at 200 *g* for 5 minutes, and the supernatants was discarded. Complete medium (Low‐DMEM mixture 10% FBS and 1% penicillin‐streptomycin) was also used to resuspend the cell pellets. BMSCs were isolated using density gradient centrifugation. Then we incubated the cells in a sterile flask (5 × 5 cm; Corning) at a cell culture chamber (37°C, 5% CO_2_). After 3 days of culture, the medium was replaced with new culture medium and the unattached cells were discarded. After the cell's reached satisfactory growth, then cells were washed with PBS, the cells were digested using trypsinase. Three lineage differentiations method was used to identify the BMSCs. Detailed on the specific method are in our previous study.^25^

#### 2.2.2 Osteogenic differentiation of BMSCs

After cultured for 21 days with osteogenic media (Cyagen), the BMSCs were harvested for mineralization assay. Firstly, BMSCs were washed with PBS three times and fixed with paraformaldehyde (4%) for 30 minutes, then incubated with 0.2% Alizarin red staining (Beyotime) for 3 minutes. Olympus inverted microscope (Olympus) was used to observe the intensity of Alizarin red staining.

#### 2.2.3 Adipogenic differentiation of BMSCs

For adipogenesis differentiation, the BMSCs were incubated with the adipogenesis‐inducing medium A (Cyagen) for 3 days, then this medium was replaced with the adipogenesis‐inducing medium B (Cyagen) continuing incubation for two more days, then was replaced back with medium A. After repeating this process for at least three times, Oil Red O was used to stain the cells for 30 minutes.

#### 2.2.4 Chondrogenic differentiation of BMSCs

For evaluating chondrogenic differentiation, the cells were put into a 15‐ml sterile tube to be washed with chondrogenesis‐inducing media (Cyagen) and centrifuged at 250 *g* for 5 minutes. Then the cells were also suspended again with chondrogenesis‐inducing medium, and centrifuged again in the same conditions, then they were resuspended again in chondrogenesis‐inducing medium. The cap of the tube was maintained loosened and kept upright inside the incubator. The medium was being refreshed gently every 3 days for three weeks. Finally, the cell pellets were carefully collected, paraffined, and stained with Alcian Blue.

#### 2.2.5 Dexamethasone treatment

After the third‐generation of BMSCs had reached satisfactory growth, we divided the cells into two groups. In group one, different concentrations (0, 10^−9^, 10^−8^, 10^−7^, 10^−6^ and 10^−5^ mol/L) of Dex were respectively added to BMSCs and kept for 4 days. The proteins were then extracted and examined by Western blot method. We selected the correct dose of Dex (10^−6^ mol/L) where Cx43 expression was significantly decreased in order to be used later. In group two, 0 or 10^‐6^ mol/L of Dex were added to the cells for 0, 1, 2, 3, 4, 5, 6, and 7 days to investigate the expressions level of Cx43, Runx2, ALP, COLⅠ, PCNA, CDK4 and p‐ERK1/2.

#### 2.2.6 CCK8 assay

To assess proliferation, cells of two groups were seeded on 96‐well plate at a density of 2 × 10^3^ cells per well and treated with or without Dex for 0, 1, 2, 3, 4 days, and we added 10 μL dye solution of Cell Counting Kit‐8 (CCK8; DOJINDO) into each well, respectively. After 3 hours, we examined the optical density of each well at 450 nm based on the manufacturer's instruction. An average optical density of 5 wells was considered as one data point.

#### 2.2.7 Immunofluorescent staining

Bone marrow stem cells of the two groups were cultured and adhered on small coverslips, then treated with or without 10^‐6^ mol/L Dex for four days. PBS solution was used to wash the cells for 5 minutes each, then fixed with paraformaldehyde (4%) for 30 minutes, and washed again with PBS for 5 minutes. The cells were penetrated with 0.3% Triton X‐100 for 20 minutes, and washed with PBS for 5 minutes each group. Cells were kept for 30 minutes in 10% goat serum at 37°C, then were all simultaneously incubated with either both Cx43 (rabbit, 1:1000; abcam) and Runx2 (mouse, 1:300; Boster Bio) or both Cx43 and ALP (mouse, 1:300, Proteintech) for overnight at 4°C. 12 hours later, the cells of both groups were washed with PBS for three times, and then secondary antibodies (FITC‐conjugated anti‐mouse, 1:300; AlexaFluor 488‐conjugated anti‐rabbit, 1:300) were added for incubation for two hours at room temperature. Finally, DAPI was used to stain the cell nucleus for 10 minutes at room temperature. Fluorescence microscope (Zeiss, Carl Zeiss) was used to obtain the images of the cells.

#### 2.2.8 Cell protein extraction and detection

RIPA buffer (Beyotime Biotechnology) with 1 mmol/L PMSF was used to lyse BMSCs on ice for 30 minutes. Then, lysates mixture was centrifuged for 15 minutes (4°C, 14 000 rpm). BCA Protein Assay kit was used to measure the proteins concentrations. The Western blot procedures were similar to those in method 2.1.4.

#### 2.2.9 Transfection assay

Two groups of lentiviral plasmids carrying either the green fluorescent protein (GFP) and Cx43 (Lv‐Cx43 group) or GFP and negative control (Lv‐NC group) were established and the materials were purchased from GeneChem (GeneChem). Third generation of BMSCs was transfected with either Lv‐Cx43 or Lv‐NC based on the manufacture's protocol. The transfected cells were named either Lv‐Cx43 or Lv‐NC, whereas the non‐transfected cells were labelled as control. 72 hours after transfection, the cells were incubated with puromycin in order to select stable transgenic cells for following studies. Fluorescence microscope was used to observe and assess the transfection effects of Lv‐Cx43. Western blot assay was also carried out to assess the overexpression effects of Lv‐Cx43 after transfected to BMSCs. The cells of Lv‐Cx43 group was pre‐treated with basic culture medium low‐DMEM with or without 5 μM PD98059 (a specific ERK1/2 inhibitor; Gene Operation) for 2 hours prior to the transfection.

#### 2.2.10 EdU assay

The proliferation of BMSCs was assessed using 5‐ethynyl‐29‐deoxyuridine (EdU) assay kit (Ribobio). Culture medium with 50 M EdU was used to incubate either the 2 days Dex‐treated BMSCs or the 4 days Dex‐treated BMSCs for one hour. Thereafter, the cells were fixed with 4% paraformaldehyde for 30 minutes and penetrated with 0.2% Triton X‐100 for 20 minutes. After being washed with PBS twice, Apollo^®^ staining working solution was added and the cells were incubated for 30 minutes at room temperature. Finally, 0.2% TritonX‐100 solution was used again to penetrate the cells, and then PBS was used to wash the cells three times. Hoechst 33342 was used to stain the cell nucleus for 15 minutes at room temperature. Three random fields were selected under a fluorescence microscope (Zeiss, Carl Zeiss) in order to calculate the rate of EdU positive cells.

### 2.3 Statistical analysis

All data analyses were done using SPSS 22.0 software (SPSS, IBM Corporation, USA). The data were presented as mean ± SD. Statistical significance different between two groups was analysed with Student's *t* test, and one‐way ANOVA with Tukey's post hoc multiple comparison tests was used to analyse the significance different among multiple groups. A two‐tailed Spearman's rank correlation coefficient (*r*) method was carried out for a correlation analysis. Statistically significant was determined by *P* value < .05. All experiments were performed repeatedly three times.

## 3 RESULTS

### 3.1 Histopathologic changes in the femoral head

**Figure 1:**
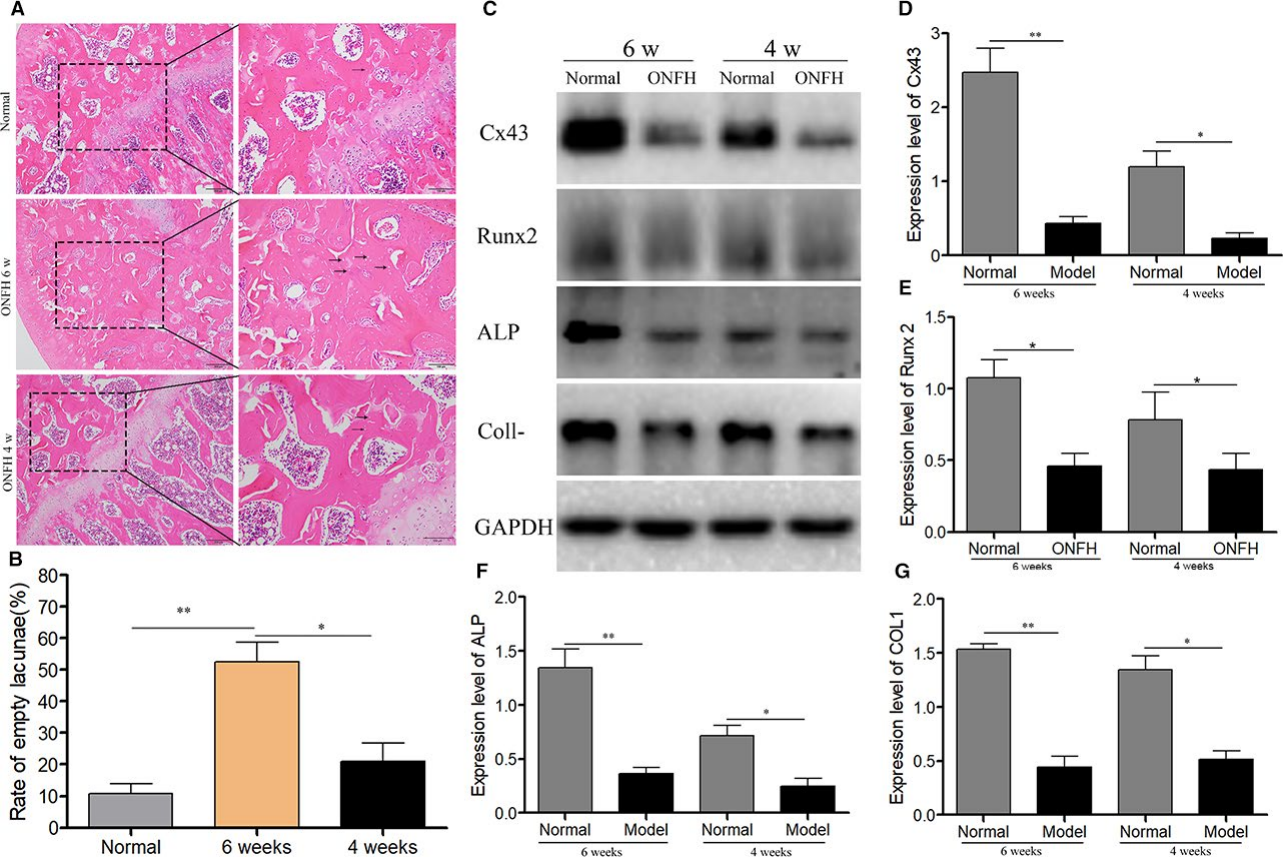
GC‐ONFH model of rats were established successfully and the expression profile of Cx43 in the femoral head. (A) The feature of histological in the normal group and the ONFH model group at 4 or 6 wks. Control group had more haematopoietic cells, few empty lacunae, lots of osteoblasts surrounding and the bone trabeculae were arrayed orderly. Empty lacunae numbers increased slightly in model group at 4 wks, whereas the model group showed typical signs of osteonecrosis at 6 wks. Scale bar indicates 200 or 100 μm. (B) Statistical analysis about the rate of empty lacunae in normal and model group. (C) The expressions of Cx43, Runx2, ALP and COLⅠ in the femoral head were examined by Western blot after the first MPS injection at 4 and 6 wks. (D‐G) Statistical analysis of Cx43, Runx2, ALP and COLⅠ expressions in femoral head in both normal and model groups at 4 and 6 wks. The data were presented as the mean ± SD; n = 3, *P < .05, **P < .001 compared with the control group. ALP, alkaline phosphatase; BMSC, bone marrow–derived mesenchymal stem cell; COLⅠ, collagen type Ⅰ; Cx43, connexin43; ONFH, osteonecrosis of femoral head; SD, standard deviation

H&E staining method was used in this study to detect empty lacunae in the bone trabeculae and determine the diagnosis of osteonecrosis. We found that control group had more haematopoietic cells, few empty lacunae, lots of osteoblasts surrounding and the bone trabeculae were arrayed orderly (Figure 1A). Whereas the model group showed typical signs of osteonecrosis such as large number of empty lacunae filling the bone trabeculae (Figure 1A) and bone trabeculae arraying disorderly, some even destroyed (Figure 1A). The empty osteocytes lacunae rate was (10.80 ± 5.38) % in the control group, and (20.98 ± 9.96) % in the model group at 4 weeks, while it was (52.4 ± 10.78) % in the model group at 6 weeks, which was elevated significantly in a comparison with the control and model groups at 4 weeks (Figure 1B). Therefore, the GC‐ONFH model of rats was successfully established based on these results.

### 3.2 Cx43 expression profile in the femoral head

There was a detection of osteogenesis related proteins in both the model and control groups (Figure 1C). Cx43 expression was significantly decreased in the model group at 6 weeks compared with the control group (Figure 1D). Additionally, the expressions of Runx2, ALP and COLⅠ were significantly decreased in the model group at 6 weeks compared with the control group (Figure 1E‐G). These findings demonstrated that the expression of Cx43 was positively correlated with the expressions of Runx2, ALP and COLⅠ after osteonecrosis in vivo, which indicated that Cx43 might play an important role in decreasing the osteogenic differentiation that occurred in GC‐ONFH.

### 3.3 The morphologic and multilineage induction of BMSCs

**Figure 2:**
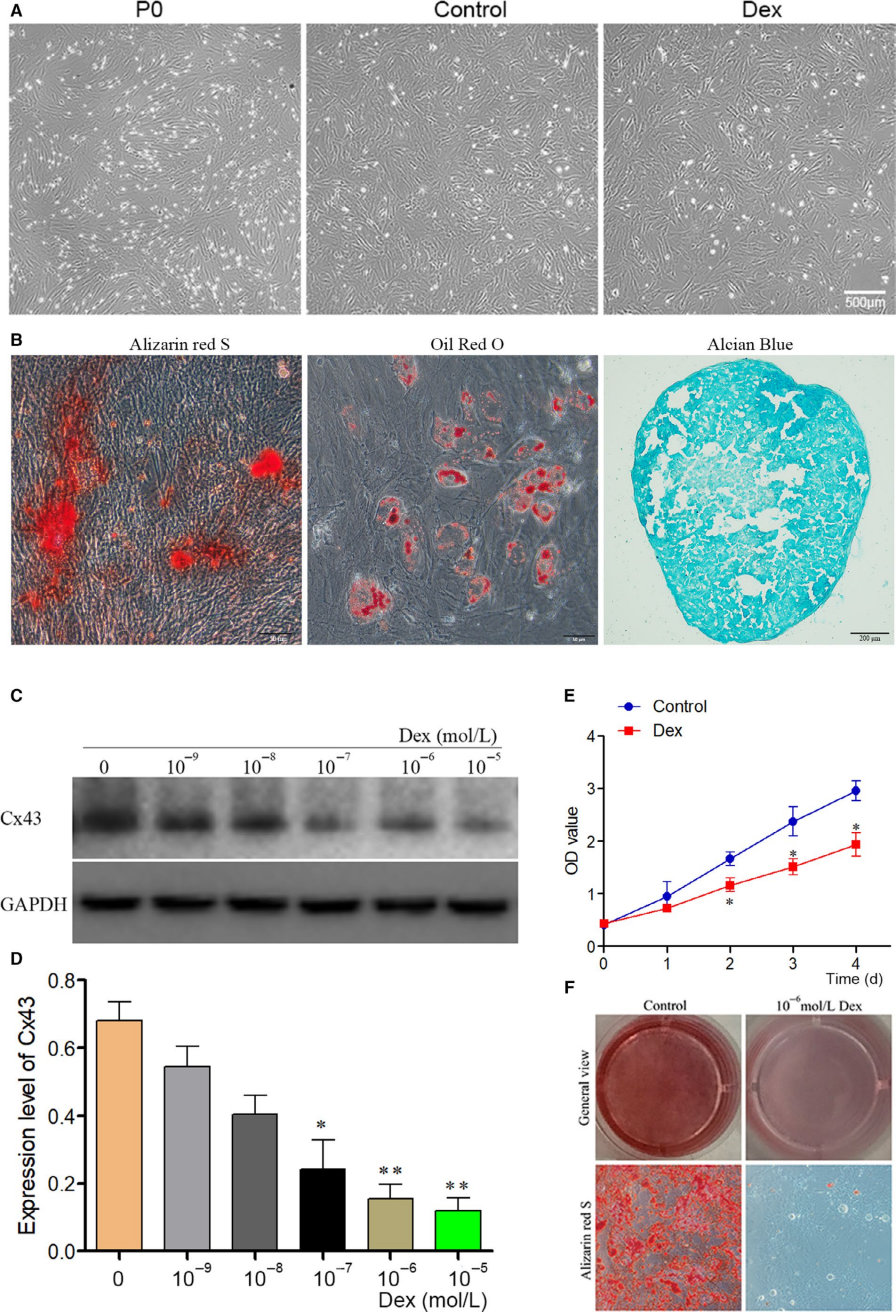
The morphological observation and identification of rat BMSCs, and the appropriate concentration of Dex on BMSCs. (A) BMSCs' morphology; primary BMSCs were spindle‐shaped (P0 and Control), cells turned into a spherical morphology after treated with Dex (Dex). (B) Multilineage induction of BMSCs. Mineralized nodules stained with Alizarin red staining; adipocytes stained with Oil Red O staining. Pellet test showed chondrogenic differentiation stained with Alcian Blue. (C) BMSCs were treated with different concentrations (0, 10^−9^, 10^−8^, 10^−7^, 10^−6^, 10^−5^ mol/L) of Dex for 4 d, and Cx43 expression was examined by Western blot to determine the most appropriate dose of Dex. (D) Statistical analysis of Cx43 expression in BMSCs after induced by different concentration of Dex. (E) BMSCs' proliferation ability after treated with 10^‐6^ mol/L Dex compared with untreated, examined by CCK8 assay. (F) Calcium nodule after BMSCs treated with 10^‐6^ mol/L Dex determined using alizarin red staining (×200); the number of calcium nodules in BMSCs; The data were presented as the mean ± SD; n = 3, *P < .05, **P < .001 compared with the control group. BMSC, bone marrow–derived mesenchymal stem cell; Dex, dexamethasone

Bone marrow stem cells of third or fourth generation proliferated quickly and reached 95% of confluence at 10 days of incubation. Their appearance was a typical long‐spindle‐like, with a number of formations of protruding (Figure 2A). In addition, the cells were positive for CD29 and CD90, but negative for CD34 and CD45 (Figure S1). Furthermore, BMSCs were identified by three lineage differentiations; our results showed that BMSCs had the ability of osteogenic differentiation, adipogenic differentiation and chondrogenic differentiation, which were verified respectively by Alizarin red staining, Oil red staining and pellet test (Figure 2B). These results demonstrated that BMSCs were isolated and cultured successfully.

### 3.4 Cx43 expression decreased in Dex‐treated BMSCs

Isolated BMSCs were cultured and treated with Dex in vitro in order to establish the rat GC‐ONFH cell model. Dex was used to reduce the osteogenic differentiation ability of BMSCs. The results showed that the expression of Cx43 was decreased gradually as Dex concentration was being increased (*P* < .05) (Figure 2C,D). Based on these results, BMSCs were treated with 10^−6^ mol/L Dex in the next analysis to mimic the cells in GC‐ONFH in vitro. After Dex treatment, BMSCs became much larger and its primary shape changed into a spherical morphology (Figure 2A); Moreover, the proliferation ability of BMSCs was significantly decreased after treated with Dex, which was verified by CCK8 assay (Figure 2E); Furthermore, the osteogenic differentiation of BMSCs significantly decreased which verified by Alizarin red staining (Figure 2F).

### 3.5 The relationship between Cx43 expression and osteogenesis related genes

**Figure 3:**
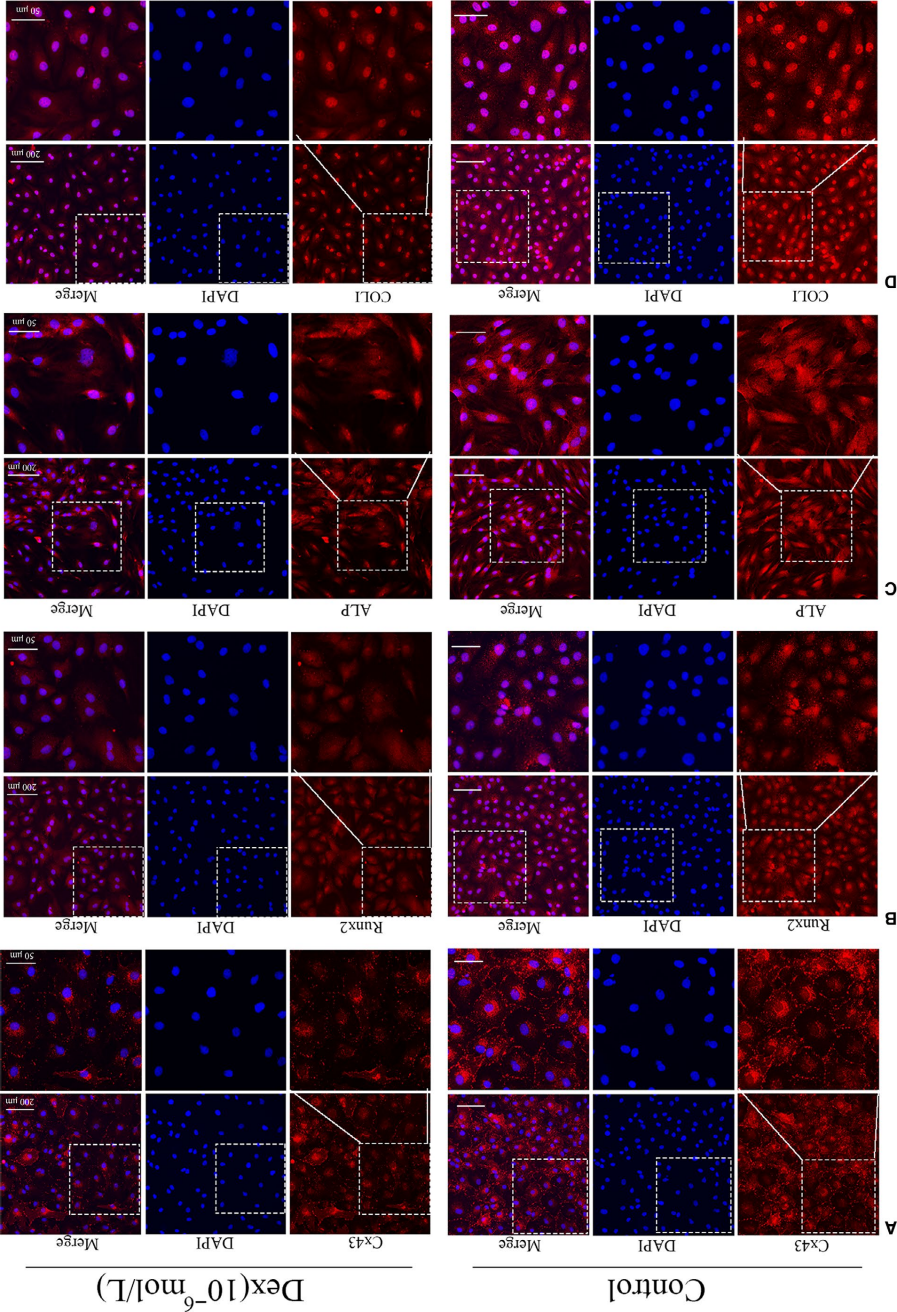
Immunofluorescence staining was used to analyse the expressions of Cx43, Runx2, ALP, and COLⅠ in normal BMSCs or Dex treatment. (A) Double immunofluorescence staining of Cx43 (red) and DAPI (blue). (B) Double immunofluorescence staining of ALP (red) and DAPI (blue). (C) Double immunofluorescence staining of Runx2 (red) and DAPI (blue). (D) Double immunofluorescence staining of COLⅠ (red) and DAPI (blue). Magnified area in the frame showed that the fluorescence intensity of Cx43, Runx2, ALP and COLⅠ decreased obviously after induced by Dex. Scale bar indicates 200 or 50 μm. Images are representatives of at least three experiments. BMSC, bone marrow–derived mesenchymal stem cell; DAPI, 4′6‐diamidino‐2‐phenylindole

**Figure 4:**
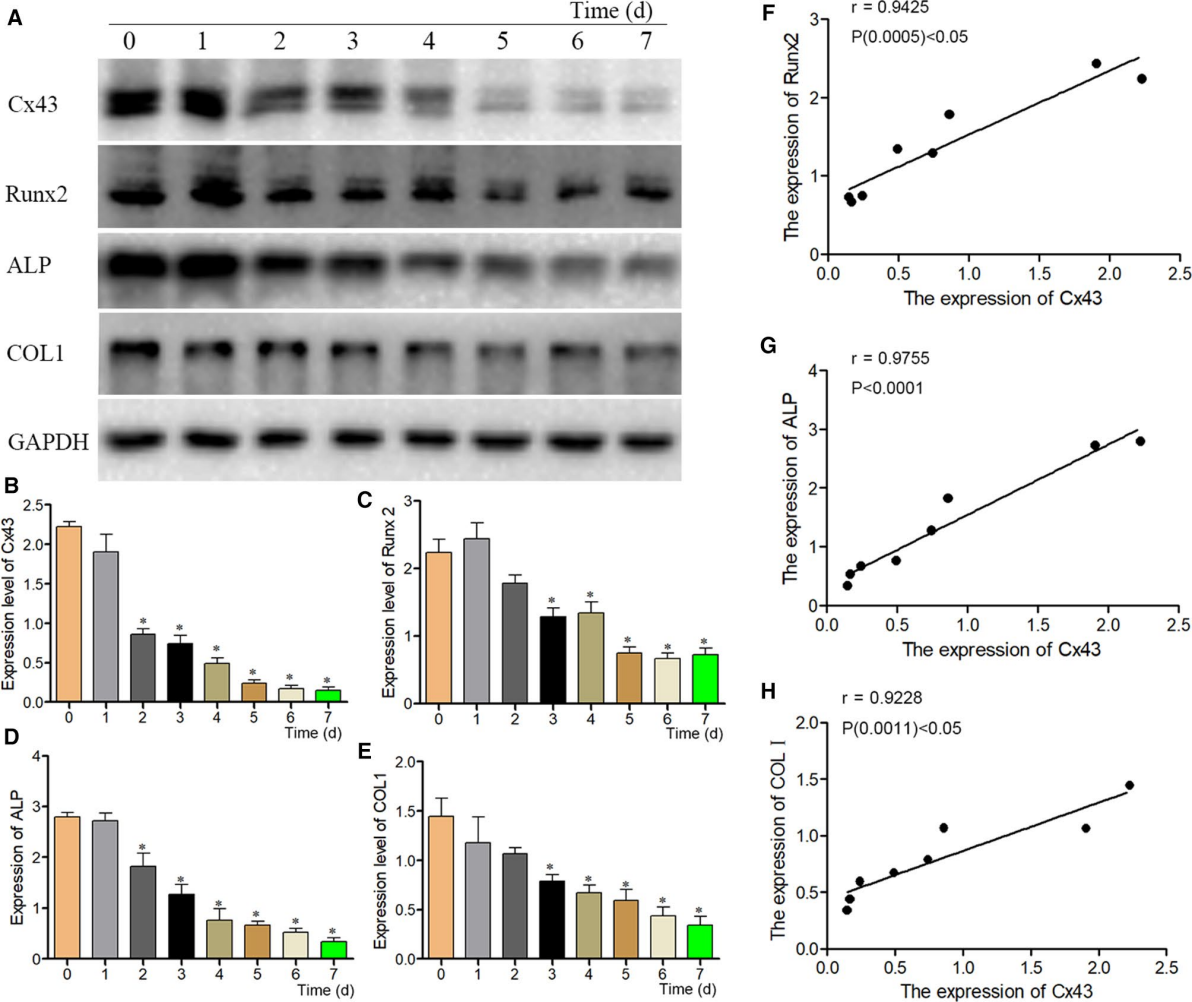
The relationship between Cx43 and osteogenic differentiation marker in BMSCs induced by Dex. (A) Cx43, Runx2, ALP, and COLⅠ were analysed by Western blot method at corresponding time point including 0, 1, 2, 3, 4, 5, 6 and 7 d after 10^−6^ mol/L Dex treatment. (B) Cx43 expression in BMSCs was analysed after treated with Dex. (C‐E) Statistical analysis of Runx2, ALP and COLⅠ expression in BMSCs after treated with Dex. The data were presented as the mean ± SD; n = 3, *P < .05 compared with the control group. (F‐H) Positive correlation between the expression of Cx43 and Runx2 or Cx43 and ALP or Cx43 and COLⅠ levels in BMSCs treated or untreated with Dex at corresponding time points by Spearman's correlation analysis

Immunofluorescence (IF) staining method was used to study the location of Cx43 in BMSCs. The expression of Cx43 was reduced obviously in BMSCs after induced by 10^−6^ mol/L Dex (Figure 3) and IFs results indicated that Cx43 expression was innately in BMSCs and Cx43 was mainly found in the cytomembrane of BMSCs (Figure 3A). The osteogenic differentiation of BMSCs was found to be inhibited by Dex treatment, therefore we assumed that Cx43 might play an important role in osteogenic differentiation of BMSCs. IFs staining was performed to observe the expressions of Runx2, ALP or COLⅠ after treated with Dex. Results showed that Cx43 expression was decreased obviously in BMSCs after treated with Dex, and the expressions of Runx2, ALP or COLⅠ were decreased as well (Figure 3B‐D). Moreover, western blot method detected the expressions of Cx43, Runx2, ALP and COLⅠ (Figure 4A). Results showed that Cx43 was significantly decreased after treated with Dex at 2 days (Figure 4B). Also the expressions of Runx2, ALP or COLⅠ were similarly decreased at the corresponding time points (Figure 4C‐E), which was mainly similar to the results of Immunofluorescence staining. Based on our correlation analysis, the expression of Cx43 was positive correlation with the expressions of Runx2, ALP or COLⅠ(Figure 4F‐H).

### 3.6 The correlation between Cx43 and cells' proliferation

**Figure 5:**
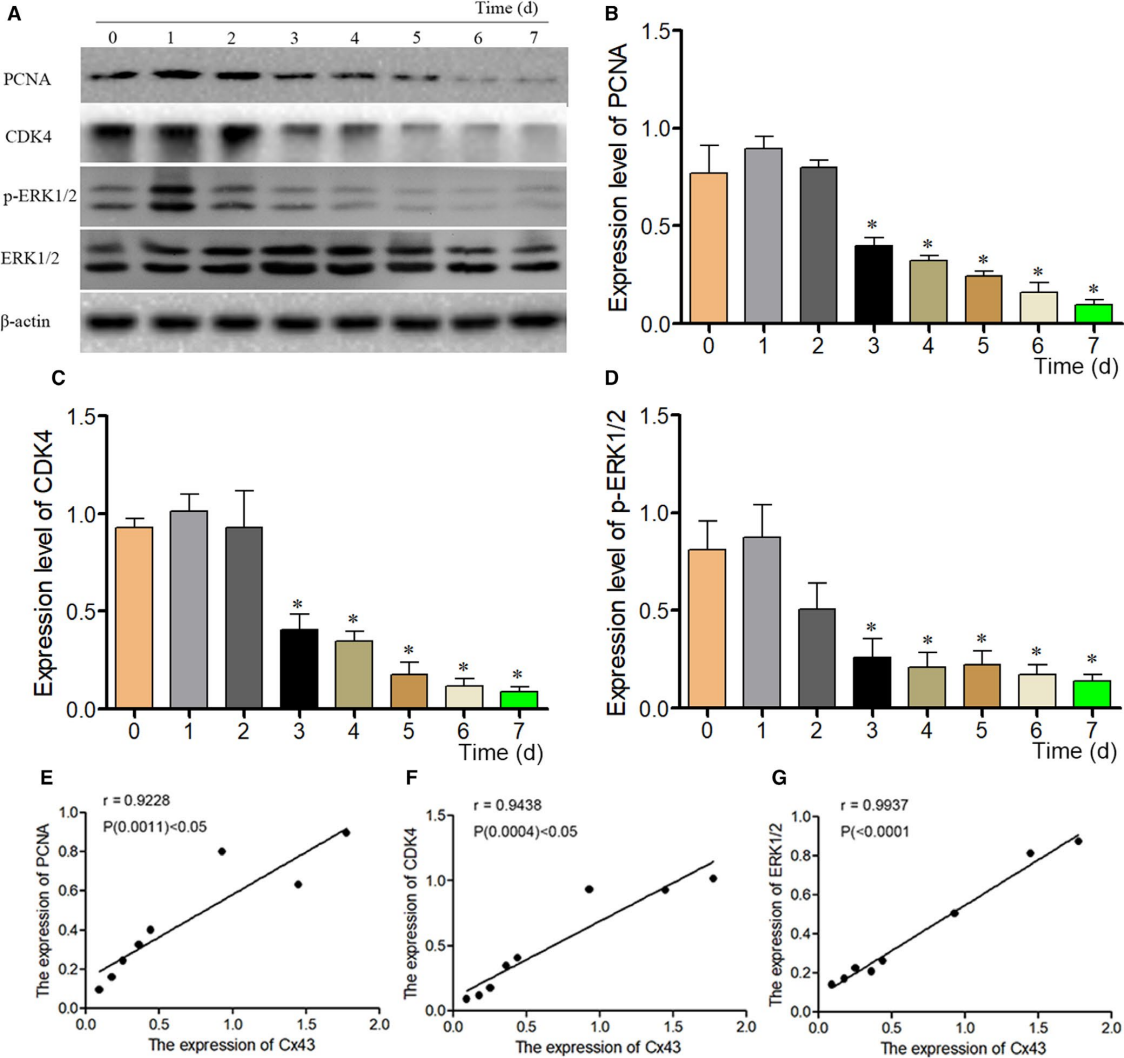
The relationship of Cx43 and specific proliferation markers. (A) PCNA, CDK4 and ERK1/2 were analysis by Western blot method at corresponding time point including 0, 1, 2, 3, 4, 5, 6 and 7 d after 10^−6^ mol/L Dex treatment. (B‐D) The expressions of PCNA, CDK4 and p‐ERK1/2 were analysis of in BMSCs after treated with Dex. The data were presented as the mean ± SD; n = 3, *P < .05 compared with the control group. (E‐G) Positive correlation between the expressions of Cx43 and PCNA or Cx43 and CDK4 or Cx43 and p‐ERK1/2 levels in normal BMSCs and Dex treated BMSCs at corresponding time points by Spearman's correlation analysis. CDK4, Cyclin‐dependent kinase4; BMSC, bone marrow–derived mesenchymal stem cell; PCNA, proliferation cell nuclear antigen

It is clear that BMSCs' proliferation is closely related to the cells' osteogenic differentiation. Therefore, it is necessary to study the correlation between Cx43 and cells' proliferation. Western blot method was used to detect the protein expressions of PCNA and CDK4 of Dex‐treated BMSCs (Figure 5A). After 3 days of culture, the expressions of PCNA and CDK4 were significantly decreased and this inhibition effects continued for 7 days (Figure 5B,C). In addition, results showed that Cx43 expression was significantly reduced in BMSCs after treated with Dex for two days. A correlation statistical analysis was carried out to investigate the correlation between Cx43 and PCNA or Cx43 and CDK4. The results showed that Cx43 expression was positively correlated with the expressions of PCNA and CDK4 in Dex‐treated BMSCs at every time point (Figure 5E,F), and the expression of p‐ERK1/2 was significantly decreased in BMSCs after induced by Dex (Figure 5D), which was positively correlated with Cx43 expression (Figure 5G). Therefore, these results indicated that Cx43 was an important molecular in BMSCs' proliferation.

### 3.7 Cx43 overexpression promoted BMSCs' proliferation and osteogenic differentiation

**Figure 6:**
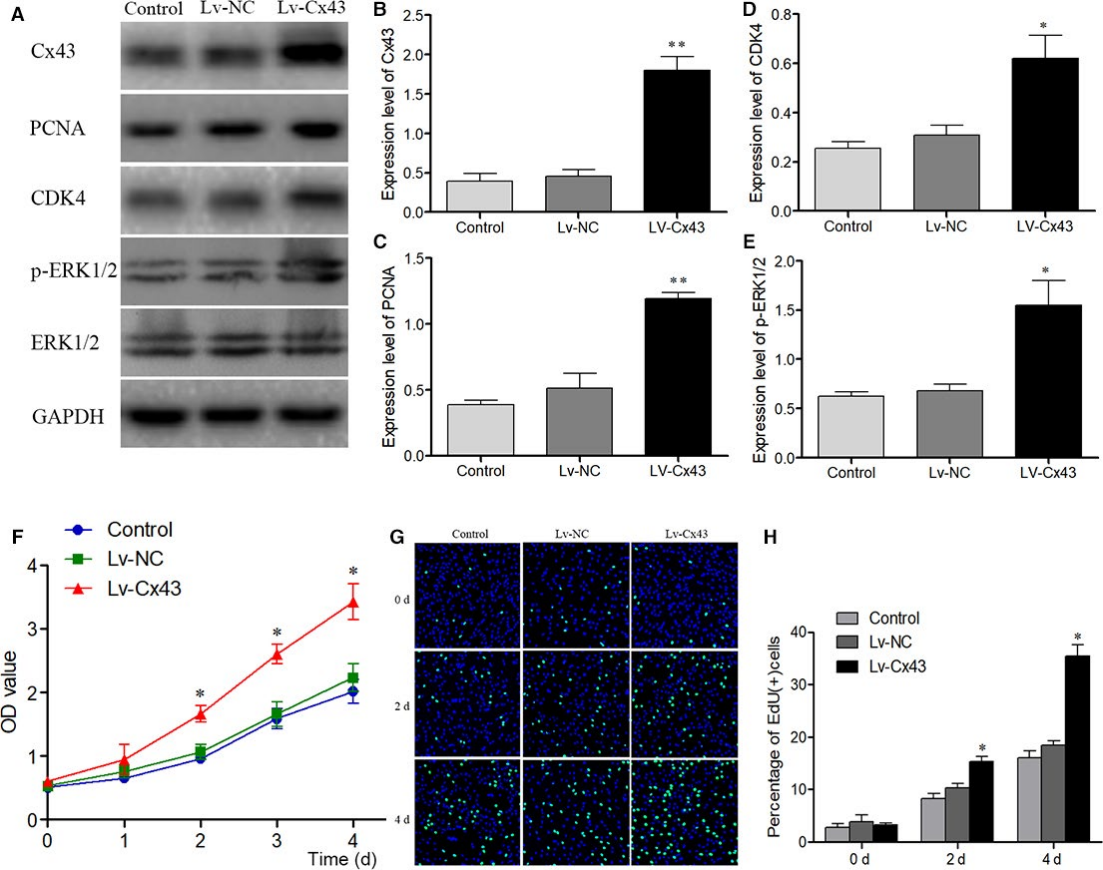
Lv‐Cx43 significantly increased Cx43 expression and Cx43 was related with BMSCs' proliferation. BMSCs were treated with Dex after transfected with Lv‐Cx43 for 72 h, and 4 days later, protein level was detected by Western blot. (A) Protein expression of Cx43, PCNA, CDK4 and p‐ERK1/2 in BMSCs after transfected with Lv‐Cx43 under the treatment of Dex. (B‐E) Statistical analysis of the ratios of PCNA/β‐actin, CDK4/β‐actin, and p‐ERK1/2/ t‐ERK1/2. The data were presented as the mean ± SD; n = 3, *P < .05, **P < 0.001compared with the control group or Lv‐NC group. (F) BMSCs' proliferation viability was assessed by CCK8 assay at 0, 1, 2, 3 and 4 d following transfection under the treatment of Dex. The data are expressed as the mean ± SD; n = 3, *P < .05 compared with the control or Lv‐NC group. (G) EdU (green) and Hoechst 33342 (blue) were double stained to assess BMSCs' proliferation after transfected with Lv‐Cx43. Scale bar indicates 200 μm. (H) Percentage of EdU‐positive cells was statistically analysed in each group for corresponding time points. The data are expressed as the mean ± SD; n = 3, *P < .05 compared with the control or Lv‐NC group. EdU: 5‐erhynyl‐2′‐deoxyuridine. BMSC, bone marrow–derived mesenchymal stem cell

**Figure 7:**
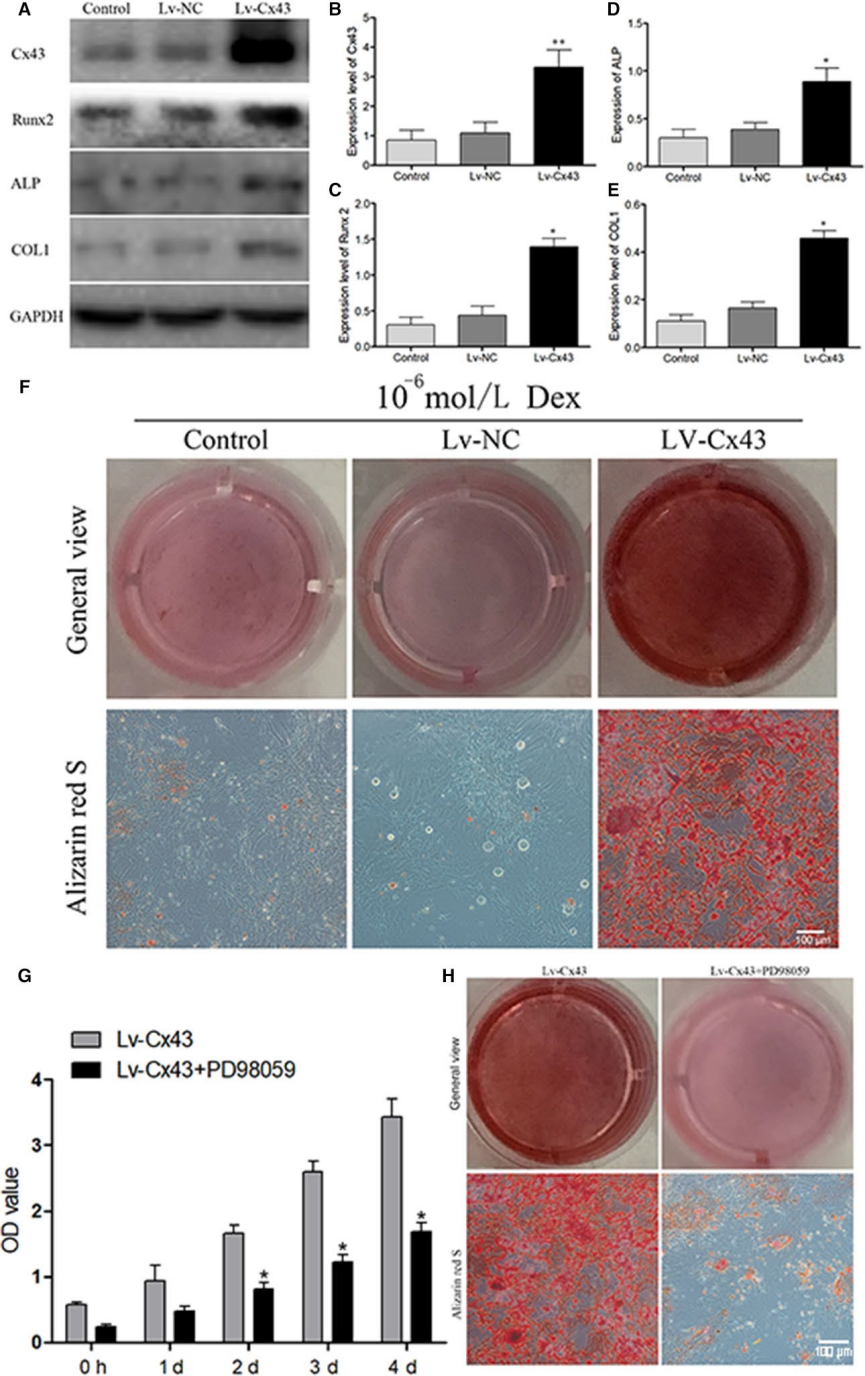
Cx43 was related with BMSCs' osteogenic differentiation. BMSCs were treated with Dex after transfected with Lv‐Cx43 for 72 h, and 4 d later, Cx43, Runx2, ALP and COLⅠ expression were detected by Western blot. (A) Protein expression of Cx43, Runx2, ALP and COLⅠ in BMSCs after transfected with Lv‐Cx43 under the treatment of Dex. (B‐E) Statistical analysis of the ratios of Cx43/GAPDH, Runx2/GAPDH, ALP/GAPDH, COLⅠ/GAPDH. The data were presented as the mean ± SD; n = 3, *P < .05, **P < .001 compared with the control group or Lv‐NC group. (F) calcium nodule was detected after transfected with Lv‐Cx43 of BMSCs under 10^‐6^ mol/L Dex treatment using alizarin red staining (×200); (G) BMSCs' proliferation ability was significantly decreased after treated with PD98059 compared with Lv‐Cx43 group; (H) the number of calcium nodules in BMSCs was obviously decreased after treated with PD98059 compared with Lv‐Cx43 group; Scale bar = 50 μm. BMSC, bone marrow–derived mesenchymal stem cell

In order to verify our hypothesis, lentivirus‐mediated Cx43 (Lv‐Cx43) gene overexpression system was established and transfected to BMSCs. The efficiency of Lv‐Cx43 was confirmed by Western blot analysis (Figure 6A). Results showed that Lv‐Cx43 significantly enhanced the expression of Cx43 compared with the control group and Lv‐NC group (Figure 6B). Moreover, the expressions of PCNA, CDK4 and p‐ERK1/2 were increased significantly after transfected with Lv‐Cx43 (Figure 6C‐E). Meanwhile, the proliferation of BMSCs was increased significantly after transfected with Lv‐Cx43 under Dex treatment, which was verified by CCK8 assay (Figure 6F) and EdU staining (Figure 6G,H). Then, the osteogenic differentiation ability of transfected BMSCs was also detected. The expressions of Runx2, ALP and COLⅠ were also increased significantly after transfected with Lv‐Cx43 under Dex treatment (Figure 7A‐E). Our results also showed that the overexpression of Cx43 had increased the number of red nodules significantly, which was verified by Alizarin red staining (Figure 7F). In addition, the expression of p‐ERK1/2 was increased after Cx43 overexpressed. Furthermore, to verify the function of the ERK1/2 pathway, we studied the effects of the PD98059 (a specific ERK1/2 inhibitor) on the effects of Cx43 on proliferation and osteogenic differentiation of the BMSCs. Our results showed that PD98059 significantly inhibited the effects of Lv‐Cx43 on promoting the proliferation and osteogenic differentiation of BMSCs, and that was identified by CCK8 assay and Alizarin red staining (Figure 7G,H). In a brief, all the results indicated that Cx43 overexpression could notably promote BMSCs' proliferation and osteogenic differentiation, and this function might be accomplished via activating ERK1/2 signalling pathway.

## 4 DISCUSSION

GC‐ONFH is a metabolic disease of the bones that is caused by multi‐factors including over dose or long term of glucocorticoids therapy.^27^, ^28^ Several possible hypotheses have been proposed explaining the pathogenesis of the GC‐ONFH, including the abnormal differentiation of BMSCs^27^, ^28^, ^29^; increased intraosseous pressure^30^; the number of fat cells^31^; fat embolism^32^; vascular thrombosis^33^; osteocytes or osteoblasts apoptosis^34^; and oxidative stress.^35^ Nevertheless, the definite pathogenesis of GC‐ONFH is still unclear.

Over the past decades, the roles that CX43 plays in the function of osteoblasts and osteocytes have been studied and figured out.^36^, ^37^ The connexins contributes to the acquisition of peak bone mass and it is a major modulator of cortical modeling.^38^ Studies demonstrated that the function of Cx43 is more than a channel, and it actively participates in generating and regulating the cell signals which could modulate skeletal development and homeostasis.^39^ It was reported that some autosomal dominant Cx43 mutations could cause oculodentodigital dysplasia (ODDD), which is a disease with craniofacial and limb dysmorphisms.^40^ Skeletal development and maintenance in post‐natal life requires the precise coordination activity of several cell types including osteoprogenitor cells, osteoblasts, osteocytes, and osteoclasts.^21^, ^38^ Therefore, understanding the molecular mechanism by which Cx43 modulates skeletal homeostasis and response to hormonal cues would be beneficial for the bone remodelling and therapeutic interventions for certain diseases. The effect of Cx43 on modulating the bone metabolism process is required in osteoblasts which accomplished though regulating bone formation and bone resorption.^41^, ^42^ However, the function of Cx43 in proliferation and osteogenic differentiation of BMSCs under Dex treatment remains unclear.

In this study, H&E staining method confirmed the successful establishment of GC‐ONFH model, and a detection of Cx43 expression was found in both GC‐ONFH model and the control group. The results showed that Cx43 expression was decreased significantly in the model group in a comparison with the control group, and also the expressions of Runx2, ALP and COLⅠ were decreased significantly in the model group. All these factors are known as the primary regulators or associated factors of the osteogenic differentiation process.^43^, ^44^ Osteocytes are mainly originated from BMSCs, where BMSCs firstly undergo lineage differentiation and become pre‐osteoblasts. Glucocorticoid use could inhibit the differentiation and maturation of pre‐osteoblasts through inhibiting Runx2 expression, which followed by a decrease of the expressions of ALP and COLⅠ.^5^, ^45^ Based on results in vivo regarding the correlation of the expression of Cx43 with the expressions of Runx2, ALP and COLⅠ, we highly speculated that Cx43 could play a functional role in the osteogenic differentiation of GC‐ONFH pathological process in vivo.

A lot of reports have demonstrated that the disease of GC‐ONFH might be closely correlated with dysfunction of BMSCs.^6^ Osteoblasts and marrow adipocytes are commonly derived from BMSCs and the metabolic balance between osteoblasts and adipocytes is very important key on maintaining the integrity of bone function.^26^, ^46^ There are many factors that could influence this balance including Dex treatment. The proliferation ability and osteogenic differentiation of BMSCs could be reduced under pathological conditions and the adipogenic differentiation increased, which could impair this metabolism balance between the cells, and result in decreasing bone mass, numbers of osteoblasts or osteocytes and reducing osteogenesis process. Recently, Kang et al have reported that high dose of Dex could inhibit the osteogenic differentiation of BMSCs.^9^

For our research, we successfully established a model of GC‐ONFH in vitro by adding high dose of Dex into isolated and cultured BMSCs from rats' bone marrow. Results showed that Cx43 expression was significantly decreased in these Dex‐treated BMSCs, and Cx43 expression was positively correlated with the expressions of Runx2, ALP and COLⅠ. Combined with the results of animal experiments in vivo, it is clearer that Cx43 is an important factor in the osteogenic differentiation of BMSCs. Furthermore, results of IFs staining showed that Cx43 protein and Runx2, ALP and COLⅠ were localized in BMSCs, and a significant decrease of fluorescence intensity of these proteins was observed in the group of Dex‐treated BMSCs. We also found that Cx43 expression was positively correlated with proliferation related proteins including PCNA, CDK4 and ERK1/2 after treated with Dex. All these results indicated that Cx43 might play an essential role in the process of proliferation and osteogenic differentiation of BMSCs. To further verify these results, we established two groups of Lv‐CX43 overexpression system and Lv‐NC plasmid, then transfected to BMSCs. After transfected with Lv‐Cx43, BMSCs' proliferation ability was enhanced under the Dex treatment, which was verified by CCK8 and EdU staining, so did the expressions of PCNA and CDK4. The expressions of Runx2, ALP or COLⅠ were also increased in BMSCs under the Dex treatment after transfected with Lv‐Cx43. In addition, Alizarin red staining results indicated that the number of calcium nodule was increased obviously. Therefore, these results suggested that there is a big relationship between Cx43 expression and BMSCs' biological properties such as proliferation and osteogenic differentiation, under the existence of Dex.

There are numbers of cells' signalling pathways that participate in regulating bone formation, and it is clear that ERK1/2 plays an important role in the process of cell proliferation,^47^ differentiation,^48^ migration,^47^ skeletal development, and osteogenesis.^49^ It is reported that the signalling pathway of ERK1/2 could initiate and arrange bone formation in a sequential cascade way; promote osteogenic differentiation of BMSCs; and inhibit BMSCs' adipogenic differentiation.^50^, ^51^ Zhang Fuqiang et al reported that platelet‐derived growth factor‐BB could promote proliferation and osteogenic differentiation of rat bone marrow stromal cells, which is mainly modulated by ERK pathway.^52^ Moreover, the effects of alendronate on BMSCs, including promoting osteogenic differentiation and inhibiting of adipogenic differentiation, are mainly mediated by ERK signalling pathway activation.^53^ Reports showed that Cx43 could significantly promote osteogenic differentiation of the cells that is derived from posterior longitudinal ligament by altering the activity of ERK.^54^ ERK1/2 also acts as a positive regulator to the osteogenic‐associated factors including Runx2, OCN, ALP and COLⅠ.^55^ Therefore, any factors that could decrease Cx43 expression might negatively regulate the ERK1/2 signalling pathway.

In this study, results showed that the expression of Cx43 was significantly decreased in the GC‐ONFH model in vivo and also in BMSCs that were induced by Dex in vitro. Our results also showed that p‐ERK1/2 expression was up‐regulated significantly after Cx43 got overexpressed, which was highly similar with the expression of PCNA, CDK4, Runx2, ALP and COLⅠ. The proliferation and osteogenic differentiation abilities were significantly increased after transfected with Lv‐Cx43, which might be complete by suppressing ERK1/2 signalling pathway. To further study the role of ERK1/2 in the process of proliferation and osteogenic differentiation of BMSCs regulated by Cx43, PD98059 (an inhibitor of ERK1/2) was used in our experiment and we observed that PD98059 significantly reduced the effects of Lv‐Cx43 on BMSCs' proliferation and osteogenic differentiation. All these findings strongly indicated that Cx43 overexpression could promote BMSCs proliferation and osteogenic differentiation significantly via activating ERK1/2 signalling pathway.

In conclusion, our results reported for the first time that Cx43 could be decreased significantly in GC‐ONFH model both in vivo and in vitro, as well as the expressions of PCNA, CDK4, Runx2, ALP and COLⅠ. In addition, the overexpression of Cx43 could enhance the proliferation and osteogenic differentiation of BMSCs. Results also indicated that Cx43 might take part in the regulation of osteogenesis process of GC‐ONFH. Finally, this study had provided important findings to be useful for further studies on the functional mechanisms and roles of Cx43 in GC‐ONFH.

## CONFLICT OF INTEREST

The authors declare that they have no conflicts of interest.

## AUTHOR CONTRIBUTIONS

**Xin Zhao:** Conceptualization (lead); Writing‐original draft (lead); Writing‐review & editing (supporting). **Mohammed Alqwbani:** Writing‐review & editing (supporting). **Yue Luo:** Formal analysis (equal). **Changjun Chen:** Formal analysis (equal). **Ge A:** Data curation (equal). **Yang Wei:** Methodology (supporting); Software (supporting). **Donghai Li:** Investigation (equal). **Qiuru Wang:** Investigation (equal). **Meng Tian:** Funding acquisition (equal); Investigation (equal). **Pengde Kang:** Project administration (lead).

## Supporting information

Figure S1

## Data Availability

The data used to support the findings of this study are available from the corresponding author upon request.
