# Supplementary figures and images for: Glucocorticoids decreased Cx43 expression in osteonecrosis of femoral head: The effect on proliferation and osteogenic differentiation of rat BMSCs
Source: J Cell Mol Med. 2020 Nov 17;25(1):484–98. doi: 10.1111/jcmm.16103 (PMC7810924; doi:10.1111/jcmm.16103)

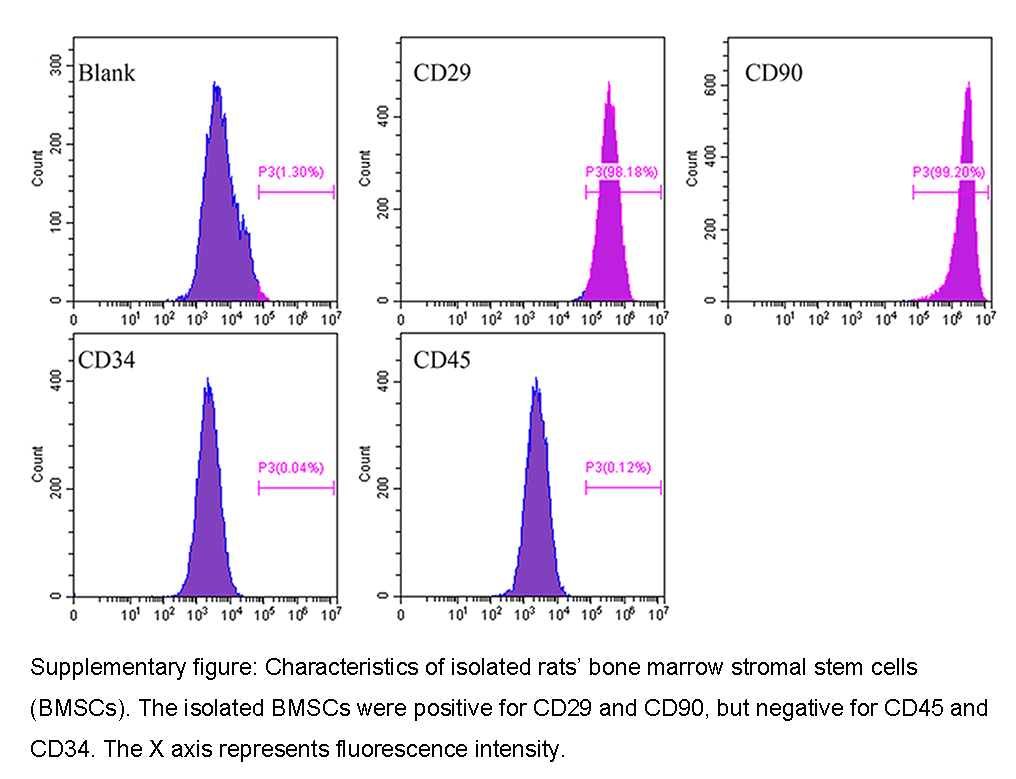

Supplement: Supplementary file 1 — Figure S1 [file JCMM-25-484-s001.tif]
